# Th2 and Th17/Th1 Inflammatory Profiles in Chronic Rhinosinusitis: Associations with Vitamin D Status and Disease Severity

**DOI:** 10.3390/ijms27136061

**Published:** 2026-07-06

**Authors:** Agnieszka Witkowska-Janik, Andrzej Wojdas, Maria Sobol, Agata Pabin, Katarzyna Komar, Ewelina Maculewicz, Piotr Rot

**Affiliations:** 1 Otolaryngology Department, Military Institute of Aviation Medicine, Krasińskiego 54/56, 01-755 Warsaw, Poland; 2 Department of Biophysics, Physiology and Pathophysiology, Medical University of Warsaw, Chałubińskiego 5, 02-004 Warszawa, Poland; 3 Department of Laboratory Diagnostics, Military Institute of Aviation Medicine, Krasińskiego 54/45, 01-755 Warsaw, Poland; 4 Clinic of Otolaryngology and Oncological Otolaryngology with Clinical Department of Cranio-Maxillofacial Surgery, Military Institute of Medicine—National Research Institute, Szaserów 128, 04-141 Warsaw, Poland

**Keywords:** chronic rhinosinusitis, type 2, cytokines, nasal polyps, interleukin, CT scoring, eosinophilic rhinosinusitis, vitamin D deficiency, 25(OH)D_3_

## Abstract

Vitamin D is believed to exert an immunomodulatory effect in the pathogenesis of chronic rhinosinusitis with nasal polyps (CRSwNP) and may influence the intensity of inflammatory processes. Therefore, this study aimed to assess serum vitamin D levels in relation to selected cytokines in patients with CRSwNP and without nasal polyps (CRSsNP). This prospective study included patients with clinically and radiologically confirmed diseases. In total, 39 patients were included in three phenotypic groups: CRSwNP (n = 17), CRSsNP (n = 10), and controls (n = 12). Serum concentrations of vitamin D (25(OH)D3), interleukins 4, 5, 17A, interferon-γ (IFN-γ), and immunoglobulin E were assessed preoperatively. The extent of subjective and objective mucosal disease was evaluated using endoscopic and radiological staging, based on the Lund–Kennedy and Lund–Mackay scoring systems in accordance with the Sino-Nasal Outcome Test-22 (SNOT-22), respectively. Patients with CRSwNP demonstrated higher serum IL-4 and IL-5 concentrations, consistent with a Th2-skewed inflammatory profile, whereas patients with CRSsNP showed higher IL-17A and IFN-γ levels, suggesting a mixed Th17/Th1 inflammatory pattern. IL-5 was positively associated with radiological disease severity. Serum vitamin D levels tended to be inversely associated with IL-5 concentration and disease severity; however, these associations did not reach statistical significance.: The findings of this study support differences in inflammatory profiles between CRSwNP and CRSsNP and confirm an association between IL-5 and radiological disease severity. Although lower vitamin D levels showed a trend toward greater inflammatory activity and disease severity, no statistically significant associations were demonstrated in this cohort. Further studies with larger populations are warranted to clarify the role of vitamin D in CRS endotypes.

## 1. Introduction

Chronic rhinosinusitis (CRS) is a long-term major inflammation of the nasal mucosa and paranasal sinuses, affecting around 10–12% of the general population when diagnosed based on either the symptoms or objective findings [1,2]. However, when both criteria are applied concurrently, the estimated prevalence decreases to below 5%, with nasal polyps being present in approximately one-third of CRS cases. The global prevalence of CRS varies considerably [3]. Although the precise prevalence of nasal polyposis (NP) remains unclear, the estimates in Europe range from 2.1% to 4.3% [4]. NP occurs more frequently in Western countries, particularly in men over 40 years of age and in patients with coexisting allergic rhinitis, asthma, and atopic dermatitis. It is associated with a substantial impairment in the quality of life as well as an increased socioeconomic burden [2]. CRS is commonly categorized into two principal phenotypes: CRS with nasal polyps (CRSwNP) and CRS without nasal polyps (CRSsNP). In Western populations, CRSwNP is typically associated with type 2 (Th2) inflammatory responses, characterized by a marked eosinophilic infiltration and elevated levels of Th2 cytokines, including IL-4, IL-5, and IL-13 [5,6]. The cytokines activate eosinophils, basophils, mast cells, and other inflammatory cells, which then induce the production of immunoglobulin E (IgE), fibrinogen, and the excessive production of mucus, resulting in the formation of nasal polyps [2]. In contrast, CRSsNP remains less clearly defined in terms of its inflammatory profile, despite representing approximately 80% of all CRS cases [7]. Inflammatory endotypes 1 and 3 are defined using representative molecular markers, including interferon gamma (IFN-γ) for type 1 (Th1) inflammation and interleukin-17 (IL-17) for type 3 (Th3) inflammation [8].

The main symptoms of CRS include rhinorrhea, nasal congestion, loss of smell, headaches, and secondary bacterial infections [5,6,9]. The chronic symptoms of CRS, particularly the loss of smell, lead to an impaired quality of life for the patients. Corticosteroid nasal sprays with saline are used for maintenance therapy [10]. Endoscopic sinus surgery (ESS) is a frequent method of removing nasal polyps and excessive mucus. However, polyp recurrence may be as high as 40% within a 2–5-year period after surgery [11]. As eosinophils constitute the majority of cells in nasal polyps, mucosal eosinophilia may be a determining factor in nasal polyp recurrence [12]. Over the past few years, the management of upper airway diseases has undergone a significant shift following the approval of monoclonal antibody-based therapies. A clinical trial evaluating the efficacy of dupilumab demonstrated that IL-4 and IL-13 were inhibited in patients with chronic sinusitis and nasal polyposis [13,14]. Yet, despite the overall good clinical response, numerous CRS patients had poor response rates to those biologics [15]. Moreover, the fact that they all come in the form of subcutaneous injections hinders their ease of use.

Previous studies have demonstrated that patients with CRS and other upper airway diseases frequently exhibit reduced serum 25 (OH)D_3_ levels [1,16]. Vitamin D is an essential nutrient that plays a central role in maintaining calcium and phosphate homeostasis. Beyond its classical functions in bone metabolism, vitamin D exerts diverse biological effects, including the regulation of cellular differentiation and proliferation, modulation of innate and adaptive immune responses, and maintenance of endocrine homeostasis [17]. Vitamin D is synthesized in the skin following exposure to sunlight and is then hydroxylated to 25-hydroxyvitamin D, the metabolite that is commonly measured in the serum–25(OH)D3 [18]. The biologically active form of vitamin D binds to the intracellular vitamin D receptor (VDR), which functions as a ligand-activated transcription factor regulating the expression of numerous genes, thereby inducing specific cellular responses. The VDR is expressed in various immune cells, including macrophages, antigen-presenting cells, and CD4^+^ T lymphocytes such as Th1, Th2, and regulatory T cells. Vitamin D was shown to suppress the production of proinflammatory cytokines, including interleukins and interferon-γ, released by T cells. Moreover, it was found to reduce the proliferation and cytotoxic activity of both T and B lymphocytes, highlighting its key role in modulating adaptive immune responses [19]. Therefore, vitamin D deficiency is associated with higher levels of inflammation in infections, allergies, and autoimmune disorders [20,21]. According to the literature, the prevalence of vitamin D deficiency in Polish respondents who were enrolled and examined through late winter and spring was 89.9% in the general adult population [22]. A review of twenty studies examining the correlation between vitamin D levels and CRS revealed that patients with CRS had lower vitamin D levels, with more pronounced deficiency being observed in those with more severe disease, particularly in cases with nasal polyps [12]. A similar correlation was found by Zand et al. [1] in a cross-sectional study of 93 patients with CRS and nasal polyps. In a study of 127 CRS patients in China, eosinophilic patients had lower vitamin D levels in the serum compared with non-eosinophilic patients, and vitamin D had the highest predictive value as an indicator of CRS [23]. Wang et al. [16] demonstrated that CRS patients with nasal polyps undergoing ESS had significantly lower serum vitamin D levels. IgE levels were inversely related to vitamin D levels in the serum. Hence, an adjunctive therapy with vitamin D supplementation may alleviate CRS symptoms [12,24]. Although previous studies have demonstrated an association between vitamin D deficiency and CRS severity, the relationship between vitamin D status and specific inflammatory endotypes of CRS remains insufficiently understood. Limited data are available regarding the associations between serum vitamin D levels, Th2- and Th17/Th1-related cytokine profiles, tissue eosinophilia, and clinical measures of disease severity. Therefore, the aim of the present study was to evaluate serum concentrations of vitamin D and selected inflammatory markers in patients with different CRS inflammatory endotypes and to investigate their associations with clinical, radiological, and histopathological indicators of disease severity. We hypothesized that lower vitamin D levels would be associated with enhanced type 2 inflammation, increased tissue eosinophilia, and greater CRS severity. By characterizing differential inflammatory patterns and their relationship with vitamin D status, this study may contribute to a better understanding of CRS endotypes and support the development of more individualized therapeutic strategies.

## 2. Results

### 2.1. Demographics of the Enrolled Patients

The study cohort comprised 39 patients who underwent surgery for chronic paranasal sinusitis. The mean age was 45 ± 14 years (range: 23–77 years), the median age was 41 years, and the IQR was 35–57 years. The cohort included 20 men (51.3%) and 19 women (48.7%). Patients were stratified into three subgroups: G1 patients operated due to paranasal sinusitis with nasal polyps, G2 patients operated due to paranasal sinusitis without nasal polyps, and G3 was the control group consisting of patients who underwent septoplasty. A statistically significant difference was observed between the study group and the sex distribution (*p* = 0.003). In G1, men constituted 52.9% (9/17) and women 47.1% (8/17). G2 included 10.0% (1/10) of men and 90.0% (9/10) of women. In G3, men constituted 83.3% (10/12) and women 16.7% (2/12). Intranasal corticosteroid use was reported in 47.1% of patients with polyps and 60.0% of patients without polyps, whereas none of the patients in the control septoplasty group used intranasal corticosteroids (0%). The prevalence of tobacco use was the highest in the polyp group (88.2%), followed by the non-polyp group (70.0%) and the control (septoplasty) group (66.7%). Osteoporosis was rare. It was observed in only one patient (5.9%) in the polyp group. Allergic rhinitis was observed in 41.2% of patients with polyps, 40.0% of patients without polyps, and 25.0% of patients in the control septoplasty group. Asthma was most common in the polyp group (23.5%) and was not observed in the control septoplasty group. Atopic dermatitis was infrequent across all subgroups. Data are presented in Table 1.

### 2.2. Comparison Between Cytokine Levels and Objective and Subjective Disease Measures

Following stratification by study group, distinct inflammatory profiles were observed. Patients with chronic rhinosinusitis with nasal polyps (G1) exhibited the lowest serum 25(OH)D3 levels together with the highest concentrations of the Th2-associated cytokines IL-4 and IL-5 and the highest eosinophil counts, consistent with an eosinophilic inflammatory phenotype. In contrast, patients without nasal polyps (G2) demonstrated the highest IL-17A and IFN-γ concentrations and the highest mean vitamin D_3_ levels, suggesting a distinct non-Th2 inflammatory profile. The control group (G3) generally exhibited the lowest concentrations of the measured cytokines, whereas total IgE levels were highest in this group. Eosinophil counts in G3 were comparable to those observed in G2. Detailed descriptive statistics for all measured variables are presented in Table 2.

Significant differences between the study groups were observed in radiological, endoscopic, clinical, and immunological parameters. Radiological and endoscopic scores differed across all groups, whereas SNOT-22 differed only between G2 and G3, and VAS scores distinguished both CRS groups from the controls. Cytokine levels also showed significant group-specific differences (Table 3). Pairwise comparisons were performed using the Mann–Whitney U test with Bonferroni correction (adjusted significance level, *p* < 0.017).

**Figure 1:**
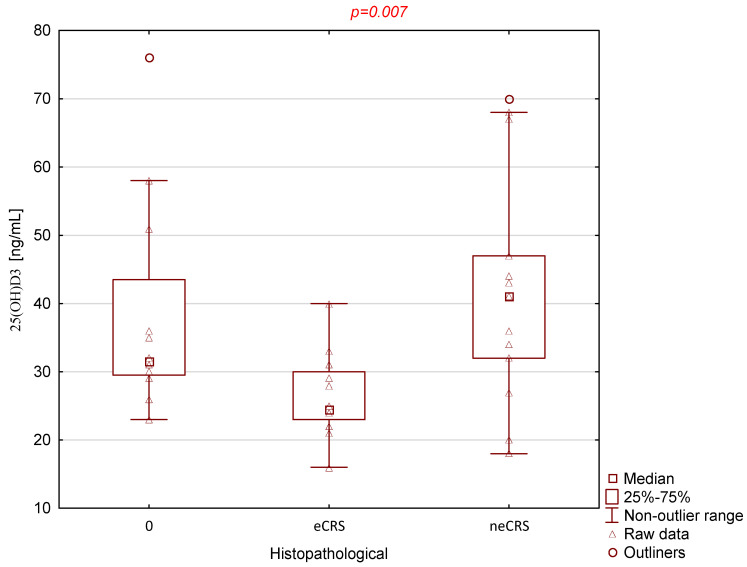
Comparison of peripheral 25(OH)D3 level according to histopathological subtype. eCRS—eosinophilic chronic rhinosinusitis (n = 12); neCRS—non-eosinophilic chronic rhinosinusitis (n = 15). The *p*-value shown in the figure was obtained using the Kruskal–Wallis test.

**Figure 2:**
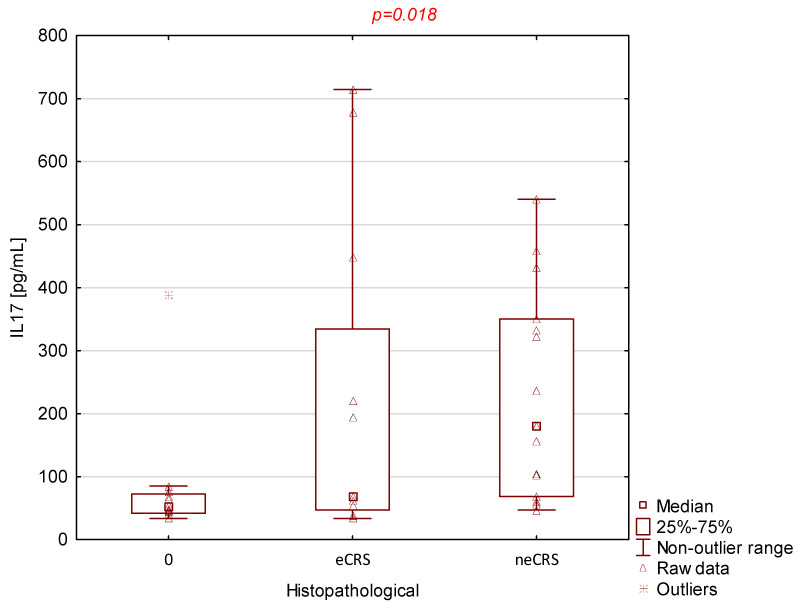
Comparison of peripheral IL-17A levels according to histopathological subtype. eCRS—eosinophilic chronic rhinosinusitis (n = 12); neCRS—non-eosinophilic chronic rhinosinusitis (n = 15). The *p*-value shown in the figure was obtained using the Kruskal–Wallis test.

**Figure 3:**
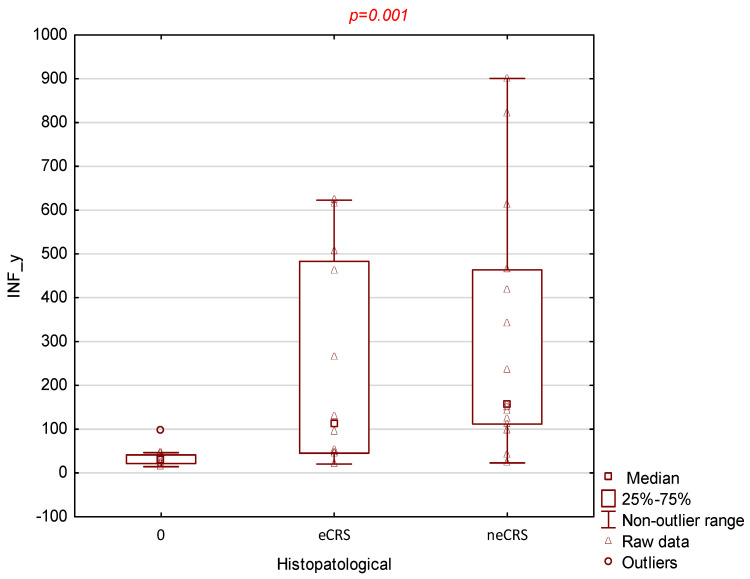
Comparison of peripheral IFN-γ levels according to histopathological subtype. eCRS—eosinophilic chronic rhinosinusitis (n = 12); neCRS—non-eosinophilic chronic rhinosinusitis (n = 15). The *p*-value shown in the figure was obtained using the Kruskal–Wallis test.

**Figure 4:**
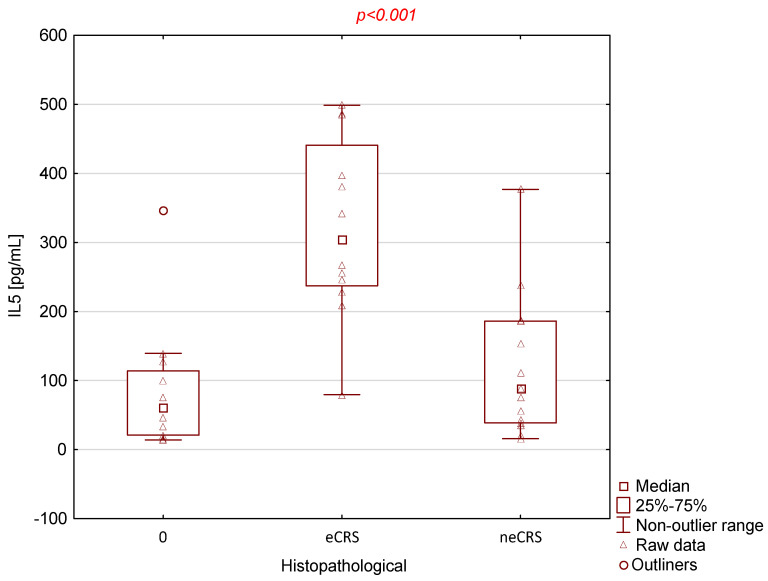
Comparison of peripheral IL-5 levels according to histopathological subtype. eCRS—eosinophilic chronic rhinosinusitis (n = 12); neCRS—non-eosinophilic chronic rhinosinusitis (n = 15). The *p*-value shown in the figure was obtained using the Kruskal–Wallis test.

These findings are further illustrated in Figure 1, Figure 2, Figure 3 and Figure 4, which show the distributions of serum 25(OH)D3, IL-4, IL-5, IL-17A, and IFN-γ concentrations across the three study groups. Patients with chronic rhinosinusitis with nasal polyps (G1) showed a predominantly Th2-associated inflammatory profile, characterized by higher IL-4 and IL-5 concentrations, whereas patients without nasal polyps (G2) exhibited higher IL-17A and IFN-γ levels. The control group (G3) generally displayed the lowest cytokine concentrations. These graphical presentations complement the statistical analyses reported in Table 3 by illustrating the magnitude and variability of the observed group differences.

Among the analyzed cytokines, IL-5 showed the most pronounced group differences. The highest IL-5 levels were observed in the eCRS group, with a median value of approximately 300 pg/mL. The interquartile range was approximately 240–440 pg/mL, while the minimum and maximum values ranged from about 80 pg/mL to 500 pg/mL, indicating markedly elevated expression of this cytokine in patients with the eosinophilic phenotype. In the neCRS group, IL-5 levels were considerably lower, with a median concentration of approximately 90 pg/mL and an interquartile range of about 40–180 pg/mL. The minimum and maximum values ranged from approximately 15 pg/mL to 375 pg/mL. The control group demonstrated the lowest IL-5 concentrations, with a median value of approximately 60 pg/mL, an interquartile range of approximately 20–110 pg/mL, and an overall range extending from about 15 pg/mL to 345 pg/mL. Consistent with these observations, IL-5 levels differed significantly between the study groups (*p* < 0.001), with the highest concentrations observed in patients with eCRS.

### 2.3. Correlations Between Cytokine Levels and Objective and Subjective Disease Measures

**Figure 5:**
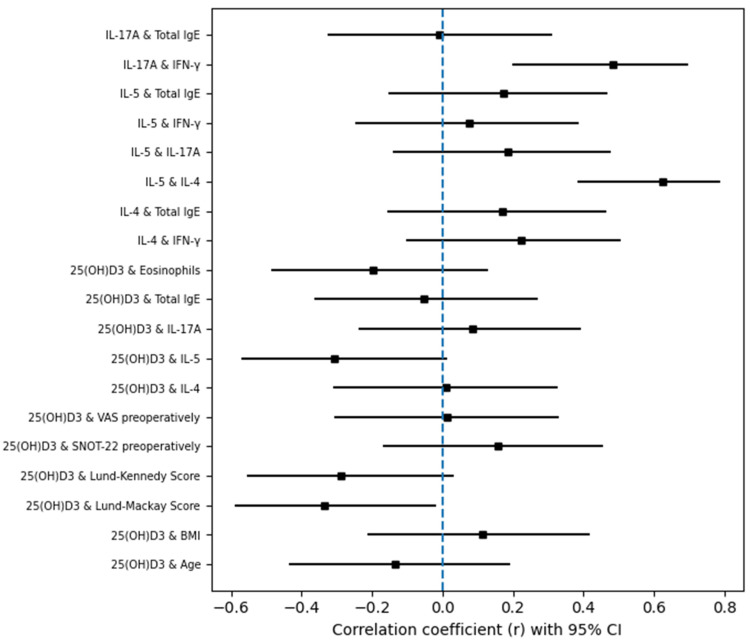
Forest plot of correlation coefficients with 95% confidence intervals. Squares indicate the correlation coefficient (r), and horizontal lines represent the 95% confidence interval.

In the overall study population, serum 25(OH)D3 levels showed a weak inverse association with the Lund–Mackay score. No significant associations were observed between 25(OH)D3 levels and the remaining clinical or laboratory parameters. Among the inflammatory markers, IL-5 was moderately positively correlated with IL-4, whereas IL-17A showed a moderate positive correlation with IFN-γ, indicating coordinated expression of these cytokines. Group-specific analyses revealed a positive correlation between IL-5 and IL-17A in G2 and a strong positive correlation between IL-4 and IL-5 in G3. Detailed correlation coefficients are presented in Table 4 and Table 5. We additionally calculated and reported 95% confidence intervals for all correlation coefficients. Standard 95% confidence intervals are presented for the overall study population (reporteded4). We also included a forest plot summarizing the correlations for the overall study population (Figure 5). For the analyses performed within the individual study groups (Table 5), which had relatively small sample sizes, 95% bias-corrected and accelerated (BCa) bootstrap confidence intervals were calculated, as appropriate.

When analyzed separately by groups (G1, G2, and G3), the following correlation was observed: in the G2 group, IL-5 was positively correlated with IL-17A (r = 0.636, *p* = 0.048). In G3, a strong positive correlation was observed between IL-4 and IL-5 (r = 0.858, *p* < 0.001).

A simple linear regression analysis was performed to evaluate the association between the Lund–Mackay score (LMS) and preoperative IL-5 concentration. Inspection of the regression diagnostics demonstrated that the assumptions of linear regression were adequately met, with approximately normally distributed residuals, no evidence of substantial heteroscedasticity, and no highly influential observations (maximum Cook’s distance = 0.184). The model was statistically significant (F (1,37) = 12.92, *p* < 0.001), explaining 25.9% of the variance in IL-5 concentrations (R^2^ = 0.259; adjusted R^2^ = 0.239). LMS was a significant positive predictor of IL-5 (β = 0.51, *p* < 0.001), with each one-point increase in LMS associated with an increase of 11.68 pg/mL in IL-5.

## 3. Discussion

Chronic rhinosinusitis comprises heterogeneous subtypes driven by diverse inflammatory mechanisms; however, its exact pathophysiology remains unclear. CRSwNP was characterized by deficient local vitamin D activation: reduced sinonasal 1α-hydroxylase and lower tissue 1,25(OH)2D3 despite similar systemic active vitamin D [25]. Lower vitamin D3 was found to correlate with more proliferating sinonasal fibroblasts, and active vitamin D reduced fibroblast proliferation in vitro [26]. The lowest mean vitamin 25(OH)D3 levels in our study were demonstrated in the group with CRSwNP, which is consistent with literature data. Multiple case–control, cohort, and meta-analytic studies showed that patients with CRSwNP had significantly lower serum 25(OH)D3 than healthy controls or CRSsNP patients [12]. Some authors suggested a vitamin D threshold (~20 ng/mL) below which an increase in the risk of polyp formation and severe disease was observed [27]. Those findings, together with recent reports, indicate a potential anti-inflammatory and immunomodulatory role of vitamin D_3_ in patients with CRSwNP [25,28,29]. In our study, the intergroup difference in vitamin D3 levels did not reach statistical significance. This finding may be attributed to the limited distribution of patients with concomitant allergic rhinitis across subgroups, which constitutes a limitation of the present study. Few small studies revealed no clear association, showing some heterogeneity [30,31,32]. Consistent with our findings, Sule et al. reported no significant differences in vitamin D_3_ levels between patients with nasal polyposis without allergic rhinitis and healthy controls [33]. A large epidemiological study by Jung Lee et al. demonstrated higher serum vitamin D3 levels in adult patients with CRS compared with individuals without CRS in the South Korean population [34]. The study was constrained by a relatively large cohort (30,609 participants), whereas our analysis was based on a small sample size, potentially allowing for a more robust evaluation.

In this cohort of 39 Polish adult participants, serum 25(OH)D3 levels did not differ significantly between patients with CRS and controls, in contrast to findings reported in previous studies. As these findings are inconsistent with previous findings, additional research in other populations is needed to better define the role of serum vitamin D in CRS. Serum 25(OH)D3 levels below 30 ng/mL were observed in only 38.46% of the participants, a finding that may be influenced by limited sunlight exposure and predominantly indoor lifestyles in Poland. Several factors may underlie the inconsistent results reported for the relationship between serum 25(OH)D3 levels and CRS in this study. Population heterogeneity, including differences in ethnicity and sunlight exposure across geographic regions, may influence the observed associations. Importantly, our study also incorporated multiple demographic variables, such as age, sex, and allergic rhinitis, that may affect vitamin D status. Another key issue is the lack of uniformity in CRS definitions, as studies by other authors applied different diagnostic criteria, ranging from individual clinical and radiographic assessments to established guidelines [16,25]. The use of an endoscopy-based score (the Lund–Kennedy score) with objective findings in CT (the Lund–Mackay score) in our study may therefore provide a more generalizable framework. Although sex-related differences in vitamin D metabolism have been reported [35,36], our additional sex-stratified analyses did not demonstrate a significant effect of sex on the observed between-group comparisons. The lack of significant correlations within individual CRS subgroups may be partly explained by the relatively small sample sizes, which limited the statistical power to detect weak-to-moderate associations. Secondly, serum vitamin D levels may not accurately reflect local vitamin D activity within sinonasal tissues, where immune responses are regulated by local expression of vitamin D receptors and vitamin D-metabolizing enzymes. The literature supports a partial disconnect: serum 25(OH)D3 is often lower in CRS or CRSwNP, but local sinonasal vitamin D activity also depends on tissue CYP27B1, CYP24A1, VDR expression, and epithelial conversion of 25(OH)D to 1,25(OH)2D, leading to the conclusion that local regulation may be independent of serum levels [16,29,37]. Additionally, the inflammatory burden in CRS is influenced by numerous interacting factors, and the contribution of vitamin D may be insufficient to produce a measurable linear relationship with individual cytokines or clinical severity indices [38]. Another possible explanation for the lack of significant associations between serum vitamin D3 levels and clinical or inflammatory parameters is that the relationship may be non-linear rather than continuous. Biological effects of vitamin D are likely to be most pronounced in individuals with vitamin D deficiency, whereas variations within the insufficient or sufficient range may have limited clinical relevance [16]. In other words, vitamin D deficiency (<20 ng/mL) may exert measurable immunomodulatory effects, while differences between moderately higher concentrations (e.g., 25–35 ng/mL) may not substantially influence disease activity. Consequently, correlation analyses based on continuous variables, such as Pearson’s or Spearman’s coefficients, may fail to detect threshold-dependent relationships. Future studies evaluating vitamin D status according to clinically relevant deficiency categories rather than absolute serum concentrations may provide additional insight into its role in CRS pathophysiology.

Interestingly, although no significant correlations were observed between serum 25(OH)D3 and most inflammatory or clinical parameters, a weak inverse correlation with Lund–Mackay score was identified in the overall cohort. This finding suggests a potential association between lower vitamin D status and greater radiological disease burden. However, the absence of similar correlations within individual CRS subgroups indicates that this relationship should be interpreted cautiously and requires confirmation in larger cohorts. Some research suggested that lower levels of 25(OH)D3 correlated with higher CT/endoscopy scores, larger polyp size, and poorer syndrome [12,29,30]. Bavi et al. [29] found an inverse correlation between serum vitamin D3 level and the LMS, LKS, and SNOT-22. Zand et al. [1] as well as Schlosser [39] observed a similar inverse correlation between vitamin D3 and the LMS and SNOT-22. Consistent with those findings, Mulligan et al. demonstrated a negative association between vitamin D3 and the extent of bone erosion on CT scans in patients with chronic and fungal rhinosinusitis [25]. In contrast, a study by Wang et al. showed no associations between the severity of the LMS and vitamin D3 level, although a negative association was identified between serum vitamin D concentrations and the polyp grade [34]. In a study by Christensen et al. [40], no significant relationship was observed between the symptoms of CRS based on the total nasal symptom score and vitamin D3 level. However, the study included 10 patients with CRSwNP.

Even though our results do not answer the question of whether vitamin D3 is inversely correlated with the LMS in CRSwNP patients, there is strong evidence to confirm that its supplementation may reduce inflammatory lesions in bones [41]. Cannell et al. evaluated the role of vitamin D3 in inflammatory disorders in patients with severe inflammation, initially presenting with high inflammatory markers and low vitamin D levels. The patients experienced modest decreases in those markers following supplementation. The findings suggest that the correction of vitamin D deficiency may contribute to a reduction in acute-phase reactants. Nevertheless, inflammatory processes may also affect vitamin D metabolism [42]. The simplest answer is that vitamin D is inversely correlated with Lund–Mackay score because lower vitamin D tends to accompany more intense sinonasal inflammation, especially eosinophilic/polyposis disease, which produces worse CT opacification [26].

Vitamin D is generally associated with the suppression of Th2 inflammation, particularly via reduction in IL-5. However, its influence on IL-4 appears to be more complex, as it may also stimulate IL-4 production under certain conditions. Therefore, while an inverse relationship is observed for IL-5, the association with IL-4 remains context-dependent [43,44]. The cytokines IL-4 and IL-5 are critical mediators of the initial stage of type 2 inflammation, contributing to eosinophil recruitment, IgE synthesis, and enhanced mucus production [45]. Not only were the serum levels of IL-4 and IL-5 the highest in G1, but they were also significantly higher than in the control group. There was also a significant difference in the level of IL-5 with an insignificant difference in the level of IL-4 in disease groups (G1 and G2). We noted a strong, positive correlation between IL-4 and IL-5 in the overall population as well as in group G3. We also observed a positive correlation between IL-5 and IFN-γ in G2. Shrestha et al. showed a significantly lower level of IL-4 in primary and recurrent NP with control groups [46] versus published data by Rai and Scavuzzo [47,48]. Such findings were not universally observed, as IL-4 levels may be affected by vitamin D deficiency and the presence of fungal infection. Lower IL-4 levels may reflect a suppressed Th2-driven response in the pathogenesis of CRSwNP [49]. Circulating IL-5 was investigated in CRS, with consistently higher levels observed in CRSwNP compared with control groups and, frequently, to CRSsNP. Evidence suggests correlations with radiologic and clinical severity, as well as with postoperative outcomes. Nonetheless, biomarker studies predominantly focused on the tissue and nasal secretion of IL-5 rather than systemic measurements [50,51]. In a study by Zielińska-Bliźniewska et al., the level of IL-5 was significantly higher in CRSwNP than CRSsNP and controls, with no difference occurring between allergic vs. non-allergic CRSwNP [50]. Du et al. assessed serum IL-5 levels before and after ESS. A significant postoperative decrease in IL-5 was observed in patients with concomitant asthma, whereas changes in non-asthmatic patients were less pronounced [51]. The use of medications prescribed by primary care physicians or taken independently by patients prior to this study enrollment may have contributed to variability in serum systemic biomarker levels.

The markedly elevated serum IL-5 levels observed in patients with eCRS are consistent with the concept that type 2 inflammation plays a central role in the pathophysiology of eosinophilic chronic rhinosinusitis. IL-5 is a key cytokine responsible for eosinophil differentiation, activation, and survival, which explains the prominent eosinophilic infiltration characteristic of this CRS phenotype [52]. Kubota et al. reported significantly higher local IL-5 concentrations in sinonasal mucosal tissue from patients with eCRS, whereas IL-5 expression was absent in patients with non-eosinophilic CRS [53]. Although local IL-5 expression was not assessed in the present study, our findings are consistent with previous reports demonstrating increased tissue IL-5 expression in eCRS.

Current literature identifies several subpopulations of helper T lymphocytes, including Th0 (naive cells), Th1, Th2, Th9, Th22, as well as the Th17 subset [54]. However, Th17 cells are not the sole source of IL-17, as this cytokine is also produced by monocytes, neutrophils, natural killer cells, CD8+ cytotoxic T lymphocytes, γδ T lymphocytes, and regulatory T cells under inflammatory conditions [55]. Conversely, a range of inhibitory signals was identified that suppressed Th17 cell activity, notably including cytokines such as IL-4, IL-5, IL-12, IL-27, and IFN-γ [56]. CRSsNP is immunologically heterogeneous. IL-17A is therefore relevant in a proportion, but not all, of CRSsNP patients. Klingler et al. showed that CRSsNP might be of Th1, Th2, or Th3 (IL-17–high). Th3 CRSsNP is characterized by IL-17A/IL-17F upregulation, acute inflammatory response, complement activation, and neutrophil-associated genes with neutrophilia rather than eosinophilia [57]. Currently available evidence indicates that the IL-17 family cytokines, particularly IL-17A and IL-17F, are crucial for host defense against bacterial and fungal pathogens. At the same time, elevated IL-17A production was linked to the pathogenesis of allergic, inflammatory, and autoimmune conditions, including psoriasis, rheumatoid arthritis, and systemic lupus erythematosus [58]. A broader mechanistic review confirmed that both CRSsNP and CRSwNP might show Th3/Th17-skewed patterns, particularly in some geographic populations, with IL-17 cytokines disrupting epithelial barrier function [59,60].

In the present study, IL-17A concentrations were the highest in the group with chronic rhinosinusitis without nasal polyps (G2) and were significantly higher compared with both G1 and G3 groups. We also identified a positive correlation between IL-17A and IL-5. The available study does not report a direct numeric IL-5–IL-17A correlation coefficient among CRS patients. It does report that lower plasma IL-5 accompanies coronary artery disease, while recombinant IL-5 suppresses Th17 differentiation and Th17 cytokine expression in vitro, which supports an inverse relationship rather than a positive one [61]. IL-17 family signaling (including IL-17A and IL-17E) shapes type-2 vs. Th17 balance, influencing autoimmune disease, allergy, and tissue repair. The “relationship” between IL-17A and IL-5 is therefore mainly indirect: IL-17E promotes IL-5 and type-2 immunity while restraining IL-17A-dominated Th17 responses [62]. We did not observe any correlation between peripheral eosinophil counts and IL-17A concentration. Hu et al. [63] conducted a study in Chinese patients and showed that those with CRS (with or without polyps) had a higher level of IL-17A in the nasal tissues in comparison with the control group, which was operated because of septal deviation. They also found significant positive correlations between IL-17A in patients with CRS and the LMS, VAS, and the LKS. In the present study, no correlation occurred between IL-17A and the LMS, VAS score, LKS, VAS, and SNOT-22. The difference related to the findings might stem from the fact that Hu et al. measured the tissue concentration of IL-17A, whereas our study used peripheral blood samples. In CRSwNP, serum IL-17A levels were significantly elevated compared with the controls and showed a significant positive correlation with the LMS (*p* < 0.001), as well as with SNOT-22 and polyp recurrence [64].

IFN-γ is the canonical marker used to define the T1 CRS endotype in modern CRS frameworks [60]. In the study by Hao, among patients with CRSwNP, IFN-γ expression correlated strongly with IL-17A, consistent with mixed T1/T3 states [65]. Our results demonstrate that IFN-γ levels were the highest in the CRSsNP group. This observation highlights the heterogeneity of CRSsNP, which should not be considered a purely “type 1” inflammatory condition but rather a combination of distinct endotypes. The distribution of these inflammatory patterns varies across geographic regions and according to the sampled tissue, influencing both the prognosis and therapeutic decision-making. IFN-γ is not uniformly elevated across all non-Th2 CRS; Asian CRSsNP cohorts sometimes showed dominant Th3/neutrophilic patterns with no significant IFN-γ increase [66]. While Th1 endotype was linked to IFN-γ-mediated responses, antiviral immunity, and the activation of CD8+ T cells, NK cells, and antigen-presenting cells, its prevalence in Western cohorts appeared to be lower than previously suggested [57,67].

IgE is more strongly linked to type 2 inflammation in CRSwNP than in CRSsNP, but a substantial CRSsNP subgroup also shows elevated IgE and type 2 features [2,68]. Across the literature, the most consistent distinction is that local tissue IgE is elevated in nasal polyps largely independent of serum IgE or atopy, whereas CRSsNP shows greater heterogeneity and weaker average type 2 intensity [68,69]. The relatively high serum IgE concentrations observed in the control group may be explained by the substantial interindividual variability of total IgE levels. Total serum IgE is a nonspecific marker that may be elevated in individuals with atopic predisposition, allergic rhinitis, environmental allergen exposure, or subclinical sensitization, even in the absence of CRS [70]. Furthermore, serum IgE levels do not necessarily reflect the intensity of local sinonasal inflammation. Given the relatively small sample size, the influence of a small number of individuals with elevated IgE concentrations on the group mean cannot be excluded.

The linear regression analysis demonstrated that the Lund–Mackay score was a significant predictor of preoperative IL-5 concentration, supporting an association between radiological disease severity and local type 2 inflammatory activity. However, the model explained approximately 26% of the variability in IL-5 levels (R^2^ = 0.259), indicating that a substantial proportion of the observed variation is attributable to factors not included in the present analysis. IL-5 expression is influenced by multiple biological and clinical determinants, including individual inflammatory profiles, disease heterogeneity, and other immunological pathways. Consequently, the regression model should be interpreted as demonstrating a statistically significant association rather than serving as a comprehensive predictive model. Furthermore, because of the relatively small sample size, the regression analysis was intentionally limited to a univariate model. Inclusion of additional covariates, such as age, sex, comorbidities, or other clinical characteristics, could have resulted in model overfitting and unstable parameter estimates. Future studies with larger cohorts should evaluate multivariable regression models to determine the independent contribution of LMS while accounting for potential confounding factors.

This study has several limitations. First, the sample size was relatively small, particularly within the individual study groups, and the sex distribution was disproportionate, which should be considered when interpreting our findings. A post hoc sensitivity analysis indicated that, with the available sample sizes, the subgroup analyses were sufficiently powered only to detect relatively strong correlations, whereas weaker associations may have remained undetected because of limited statistical power. Consequently, the absence of statistically significant correlations should not be interpreted as evidence of no association but rather as a reflection of the limited ability to detect small-to-moderate effect sizes. Therefore, the subgroup analyses should be regarded as exploratory and interpreted with appropriate caution. Furthermore, the modest sample size and the single-center design may limit the robustness and generalizability of the findings. Future multicenter studies involving larger, adequately powered cohorts with a more balanced sex distribution are warranted to validate our findings and confirm the observed associations. Third, tissue eosinophil counts were assessed only in CRS patients because nasal tissue samples were not collected from control subjects, limiting direct comparisons of local eosinophilic inflammation between the groups. Finally, the cross-sectional design precludes establishing causal relationships between vitamin D levels and inflammatory cytokine profiles. Another limitation of this study is that the cytokine panel was restricted to selected markers representing Th2- and Th17/Th1-associated immune responses. Although these cytokines were chosen according to the predefined study protocol, the inclusion of additional inflammatory mediators, such as IL-1β, IL-6, IL-10, IL-11, and IL-13, could provide a more comprehensive characterization of CRS inflammatory endotypes and should be considered in future research. Despite these limitations, the study provides novel insights into the association between vitamin D status and Th1-, Th2-, and Th17-related inflammatory patterns in CRS.

The relatively small sample size, particularly within the individual study groups, should be considered when interpreting our findings. A post hoc sensitivity analysis indicated that, with the available sample sizes, the subgroup analyses were sufficiently powered only to detect relatively strong correlations, whereas weaker associations may have remained undetected because of limited statistical power. Consequently, the absence of statistically significant correlations should not be interpreted as evidence of no association but rather as a reflection of the limited ability to detect small-to-moderate effect sizes. In addition, the modest sample size may reduce the robustness and generalizability of the findings. Therefore, the subgroup analyses should be regarded as exploratory, and the observed associations require confirmation in larger, adequately powered prospective studies.

## 4. Materials and Methods

### 4.1. Study Design and Patient Recruitment

This prospective study enrolled adult patients (≥18 years) in the Department of Otolaryngology who were able to provide informed consent and agreed to participate. All the participants had a diagnosis of chronic rhinosinusitis established according to the European Position Paper on Rhinosinusitis and Nasal Polypscriteria [71], exhibited the failure of conservative management defined as the persistence of symptoms for more than 12 weeks despite optimal pharmacological treatment, and presented with inflammatory lesions on sinus computed tomography (CT). The control group comprised patients scheduled for septoplasty due to nasal septal deviation. All controls had normal sinus CT findings and did not fulfill the EPOS diagnostic criteria for chronic rhinosinusitis. Sinonasal symptoms were evaluated using the SNOT-22 questionnaire. The low SNOT-22 scores observed in this group were mainly attributable to nasal obstruction related to septal deviation and non-specific quality-of-life items, rather than symptoms indicative of chronic rhinosinusitis. Disease severity was assessed using the Lund–Mackay and Lund–Kennedy scoring systems. The Lund–Mackay score is a radiological staging system based on computed tomography (CT) findings, in which each paranasal sinus is scored as 0 (no opacification), 1 (partial opacification), or 2 (complete opacification), with a maximum total score of 24 points [72]. The Lund–Kennedy endoscopic score assesses nasal polyps (0–2 points), edema (0–2 points), and nasal discharge (0–2 points) for each nasal cavity, with higher total scores reflecting greater disease severity [73]. The exclusion criteria were: the lack of informed consent, disease limited to a single sinus, ciliary dysfunction (e.g., cystic fibrosis), suspected fungal infection, poor adherence to medical therapy, suspected neoplastic or autoimmune disease (particularly granulomatosis with polyangiitis), confirmed or suspected immunodeficiency, coagulation disorders, and the use of systemic corticosteroids in the preoperative period. To minimize the potential confounding effect of orally administered vitamin D on the study outcomes, individuals taking medications or dietary supplements containing vitamin D were excluded from participation.

The symptoms were additionally evaluated using the Sino-Nasal Outcome Test-22 questionnaire (SNOT-22). It is a validated, widely used instrument for assessing quality of life. It comprises 22 items, each scored on a scale from 0 to 5, where 5 indicates the most severe symptoms. The second validation was done by the Visual Analogue Scale (VAS); higher total scores reflect poorer health-related quality of life.

### 4.2. Sample Collection

Ten milliliters of venous blood were collected from each participant by sterile venipuncture into Vacutainer tubes containing a clot activator. The samples were allowed to clot at room temperature and were subsequently centrifuged at 3000 rpm for 15 min. The resulting serum was carefully separated and transferred into sterile Eppendorf tubes and stored at −20 °C until analysis. Serum concentrations of IL-4, IL-5, IL-17A, IFN-γ were determined using commercially available ELISA kits: Human IL-4 ELISA Kit (orb50048), Human IL-5 ELISA Kit (orb50157), Human IL-17A ELISA Kit (orb50063), and Human IFN-γ ELISA Kit (orb50030) (Biorbyt Ltd., Cambridge, UK), according to the manufacturer’s instructions. Serum 25-hydroxyvitamin D3 was measured using the Elecsys Vitamin D total III competitive electrochemiluminescence binding assay on a cobas e402 analyzer (Roche Diagnostics GmbH, Mannheim, Germany). The assay had an analytical measuring range of 3.0–120 ng/mL, a limit of detection of 3.0 ng/mL, and a limit of quantification of 6.0 ng/mL. Serum 25(OH)D3 concentrations were classified as deficient (≤20 ng/mL), insufficient (21–29 ng/mL), or sufficient (≥30 ng/mL).

### 4.3. Histological Analysis

NP tissues were obtained from patients with CRSwNP, whereas mucosa from the agger nasi cell was collected during routine functional endoscopic sinus surgery in patients with CRSsNP. No tissue samples were collected in the control group. The classification of CRS phenotypes was based on tissue eosinophil density. Histopathological evaluation was performed by an experienced pathologist who was blinded to the clinical characteristics of the participants and study group allocation. Eosinophils were counted in five high-power fields (HPFs; ×400 magnification), and the mean eosinophil count was calculated for each specimen. Patients were classified as having eosinophilic CRS (eCRS) when the mean tissue eosinophil count was ≥10 eosinophils/HPF, while those with <10 cells/HPFs were considered non-eosinophilic (neCRS) in accordance with previously published criteria and EPOS 2020 recommendations [71,74].

### 4.4. Statistical Analysis

All statistical analyses were conducted using Statistica 13.0 software (Dell Software Inc., Round Rock, TX, USA) and Microsoft^®^ Excel version 16.89.1 (Microsoft Corporation, Redmond, WA, USA). Quantitative variables were summarized using descriptive statistics, including the mean, standard deviation, median, IQR, and range. The normality of each variable’s distribution was assessed using the Shapiro–Wilk test. Since the variables were not normally distributed, the Kruskal–Wallis test was used to assess whether statistically significant differences occurred for continuous variables in the groups. When the obtained *p*-value was below the predefined level of statistical significance, pairwise comparisons were performed using the Mann–Whitney U test to identify which groups differed significantly. To account for multiple comparisons, the level of statistical significance was adjusted to 0.017. Categorical variables were expressed as frequencies and percentages. Comparisons between independent categorical variables were conducted using the chi2 test or Fisher’s exact test, as appropriate. To assess the correlations between analyzed variables, Spearman’s rank correlation coefficient was calculated. The strength of correlation was interpreted as follows: very strong (0.90–1.00), strong (0.70–0.89), moderate (0.40–0.69), weak (0.10–0.39), and negligible (0.00–0.09). Assuming a two-sided significance level of 0.05 and 80% statistical power, the study was able to detect correlations of approximately |ρ| ≥ 0.43 in the overall study population (n = 39), |ρ| ≥ 0.61 in the subgroup with n = 17, |ρ| ≥ 0.70 in the subgroup with n = 12, and |ρ| ≥ 0.75 in the subgroup with n = 10.

To evaluate the association between the LMS and preoperative IL-5 concentration, a simple linear regression analysis was performed. Prior to model interpretation, the assumptions of linear regression were assessed. Normality of the residuals was evaluated using normal probability (Q–Q) plots, homoscedasticity was assessed by visual inspection of residuals-versus-fitted values plots, and influential observations were examined using Cook’s distance. Given the relatively small sample size, the regression analysis was limited to a univariate model to minimize the risk of overfitting. Therefore, additional covariates, such as age, sex, and comorbidities, were not included in the model.

## 5. Conclusions

CRS exhibits distinct inflammatory endotypes depending on the presence of nasal polyps. CRSwNP is characterized by a Th2-skewed profile with elevated IL-4 and IL-5, whereas CRSsNP shows the predominance of IL-17A and IFN-γ, suggesting a mixed Th17/Th1 response. Significant differences in clinical severity and cytokine expression between groups support the relevance of endotype-based classification. IL-5 was positively associated with radiological severity, indicating its potential as a biomarker of disease burden. Although vitamin D showed a trend toward an inverse relationship with disease severity and IL-5, these associations were not statistically robust. Moreover, the study suggests a relationship between vitamin D levels and eosinophilic inflammation. Further studies are needed to clarify the role of vitamin D in CRS.

## Figures and Tables

**Table 1:** Distribution of clinical characteristics and comorbidities according to study subgroup (polyps, without polyps, and control septoplasty group).

	G1 (n = 17)	G2 (n = 10)	G3 (n = 12)
Intranasal steroids	8 (47.06)	6 (60%)	0 (0%)
Tobacco use	2 (11.8%)	3 (30%)	4 (33.3%)
Osteoporosis	1 (5.9%)	0 (0%)	0 (0%)
Allergic rhinitis	7 (41.2%)	4 (40%)	2 (25%)
Atopic dermatitis	0 (0%)	1 (10%)	1 (8.3%)
Asthma	4 (23.5%)	1 (10%)	0 (0%)
Age			
Mean ± SD	48 ± 16	44 ± 16	41 ± 8
(Min–Max)	(23–27)	(23–63)	(29–55)
BMI			
Mean ± SD	25.7 ± 4.1	25.4 ± 2.4	27.4 ± 5.1
(Min–Max)	(18.5–32.4)	(21.6–29.3)	(20.1–40.6)
SNOT-22			
Mean ± SD	47.4 ± 23.1	56.4 ± 21.4	32.5 ± 17.9
VAS			
Mean ± SD	22.8 ± 9.5	23.4 ± 9	12.4 ± 5.9
Lund–Kennedy			
Mean ± SD	8.36 ± 1.22	4.2 ± 1.40	1.25 ± 1.22
Lund–Mackay			
Mean ± SD	13.53 ± 4.17	8.0 ± 2.75	0.34 ± 0.65

**Table 2:** Overall results of the measured parameters.

Parameter	All (n = 39) Mean ± SD Median (Q1–Q3)	G1 (n = 17) Mean ± SD Median (Q1–Q3)	G2 (n = 10) Mean ± SD Median (Q1–Q3)	G3 (n = 12) Mean ± SD Median (Q1–Q3)
25(OH)D3 [ng/mL]	35.9 ± 14.9 32.0 (25–43)	30.9 ± 12.0 28.0 (24–34)	41.8 ± 17.0 40.5 (29–47)	38.1 ± 15.6 31.5 (29.5–43.5)
IL-4 [pg/mL]	186.1 ± 202.0 142.8 (31.2–243.3)	296.6 ± 251.8 186.6 (142.8–439.9)	120.8 ± 102.3 92.9 (29.3–224.3)	84.1 ± 79.6 56.6 (20.4–121.5)
IL-5 [pg/mL]	171.1 ± 147.9 128.0 (42.6–255.8)	264.2 ± 149.1 245.7 (154.1–380.9)	116.7 ± 111.9 68.2 (38.6–228.6)	84.5 ± 93.3 60.6 (21.1–114.1)
IL-17A [pg/mL]	180.7 ± 189.5 68.6 (48–322)	172.3 ± 194.1 68.2 (56.7–194.5)	313.7 ± 200.3 284.2 (179.9–458.6)	81.9 ± 97.7 51.2 (41.9–72.5)
IFN-γ [pg/mL]	203.3 ± 244.4 97.0 (28.5–342.9)	166.9 ± 170.3 111.6 (46.9–234.2)	467.5 ± 286.5 483.1 (156.4–613.6)	34.7 ± 22.2 28.5 (21.7–41.2)
Total IgE [IU/mL]	120.0 ± 172.9 58.4 (13.5–115.9)	129.1 ± 197.0 60.4 (25.9–82.0)	78.2 ± 136.2 28.6 (13.5–68.3)	146.3 ± 170.6 73.3 (10–231)
Eosinophils [cells/µL]	4.4 ± 3.1 3.7 (2.2–5.8)	5.6 ± 3.5 4.8 (3.5–6.6)	3.4 ± 2.1 3.0 (1.7–4.8)	3.4 ± 2.9 3.0 (1.1–5.1)

**Table 3:** Comparison between groups.

	G1 vs. G2, *p*	G2 vs. G3, *p*	G1 vs. G3, *p*
Lund–Mackay Score	0.002	<0.001	<0.001
Lund–Kennedy Score	<0.001	<0.001	<0.001
SNOT-22	0.359	0.006	0.080
VAS preoperatively	0.711	0.011	0.002
25(OH)D3	0.093	0.194	0.628
IL-4	0.093	0.497	0.007
IL-5	0.011	0.456	<0.001
IL-17A	0.046	0.003	0.080
IFN-γ	0.006	<0.001	0.002
IgE	0.414	0.853	0.979

**Table 4:** Correlations among clinical and inflammatory parameters in the overall study population.

Variable	r	95% CI L	95% CI U	*p*
25(OH)D3 & Age	−0.135	−0.432	0.189	0.412
25(OH)D3 & BMI	0.113	−0.210	0.414	0.493
25(OH)D3 & Lund–Mackay Score	−0.334	−0.588	−0.021	0.038
25(OH)D3 & Lund–Kennedy Score	−0.288	−0.553	0.030	0.075
25(OH)D3 & SNOT-22 preoperatively	0.158	−0.166	0.451	0.337
25(OH)D3 & VAS preoperatively	0.012	−0.305	0.326	0.944
25(OH)D3 & IL-4	0.01	−0.306	0.324	0.952
25(OH)D3 & IL-5	−0.307	−0.568	0.009	0.058
25(OH)D3 & IL-17A	0.086	−0.236	0.391	0.602
25(OH)D3 & Total IgE	−0.052	−0.362	0.268	0.758
25(OH)D3 & Eosinophils	−0.197	−0.483	0.126	0.23
IL-4 & IFN-γ	0.224	−0.098	0.504	0.17
IL-4 & Total IgE	0.17	−0.154	0.461	0.315
IL-5 & IL-4	0.625	0.385	0.786	<0.001
IL-5 & IL-17A	0.187	−0.137	0.475	0.255
IL-5 & IFN-γ	0.076	−0.245	0.382	0.646
IL-5 & Total IgE	0.174	−0.150	0.464	0.302
IL-17A & IFN-γ	0.485	0.200	0.694	0.002
IL-17A & Total IgE	−0.008	−0.323	0.308	0.964

Spearman’s correlation coefficients (r) with corresponding 95% confidence intervals (CI) and *p*-values.

**Table 5:** Correlations among clinical and inflammatory parameters within the study groups.

Pair	G1 ρ [95% BCa CI]	G1 *p*	G2 ρ [95% BCa CI]	G2 *p*	G3 ρ [95% BCa CI]	G3 *p*
25(OH)D3 & Age	−0.037 [−0.618, 0.539]	0.888	−0.285 [−0.776, 0.455]	0.425	0.086 [−0.640, 0.790]	0.79
25(OH)D3 & BMI	0.002 [−0.580, 0.553]	0.993	−0.321 [−0.801, 0.503]	0.365	0.224 [−0.548, 0.793]	0.484
25(OH)D3 & LMS	−0.321 [−0.721, 0.233]	0.21	−0.239 [−0.764, 0.554]	0.507	−0.099 [−0.631, 0.596]	0.759
25(OH)D3 & LKS	−0.116 [−0.616, 0.456]	0.658	0.290 [−0.477, 0.838]	0.416	−0.337 [−0.819, 0.464]	0.285
25(OH)D3 & SNOT-22	0.065 [−0.505, 0.587]	0.804	0.200 [−0.610, 0.862]	0.58	0.211 [−0.513, 0.800]	0.511
25(OH)D3 & VAS	0.250 [−0.319, 0.680]	0.333	−0.042 [−0.714, 0.637]	0.907	−0.127 [−0.610, 0.580]	0.694
25(OH)D3 & IL-4	0.076 [−0.407, 0.572]	0.771	0.127 [−0.698, 0.789]	0.726	0.249 [−0.513, 0.771]	0.435
25(OH)D3 & IL-5	−0.322 [−0.742, 0.375]	0.208	−0.273 [−0.870, 0.447]	0.446	0.245 [−0.374, 0.778]	0.442
25(OH)D3 & IL-17A	−0.260 [−0.643, 0.226]	0.313	−0.115 [−0.811, 0.654]	0.751	0.553 [0.047, 0.827]	0.062
25(OH)D3 & IFN-γ	−0.201 [−0.588, 0.304]	0.438	−0.394 [−0.963, 0.610]	0.26	0.242 [−0.486, 0.805]	0.449
25(OH)D3 & Total IgE	−0.172 [−0.634, 0.327]	0.508	−0.067 [−0.623, 0.711]	0.855	0.207 [−0.706, 0.801]	0.565
25(OH)D3 & Eosinophils	−0.208 [−0.649, 0.358]	0.424	−0.115 [−0.873, 0.702]	0.751	0.095 [−0.619, 0.791]	0.77
IL-4 & IFN-γ	0.130 [−0.440, 0.665]	0.619	0.224 [−0.494, 0.731]	0.533	0.270 [−0.438, 0.816]	0.397
IL-5 & IFN-γ	−0.279 [−0.736, 0.379]	0.277	−0.212 [−0.887, 0.620]	0.556	0.021 [−0.628, 0.620]	0.948
IL-17A & IFN-γ	0.439 [−0.278, 0.833]	0.078	−0.261 [−0.774, 0.542]	0.467	−0.007 [−0.584, 0.560]	0.983
IL-17A & Eosinophils	−0.124 [−0.685, 0.516]	0.303	0.345 [−0.623, 0.863]	0.134	0.091 [−0.495, 0.636]	0.323
IL-4 & IL-5	0.414 [−0.210, 0.765]	0.098	0.539 [−0.304, 0.917]	0.108	0.858 [0.644, 0.993]	<0.001
IL-4 & IL-17A	0.252 [−0.340, 0.730]	0.328	0.442 [−0.358, 0.827]	0.2	0.504 [−0.161, 0.829]	0.094
IL-5 & IL-17A	0.027 [−0.559, 0.614]	0.918	0.636 [−0.119, 0.963]	0.048	0.497 [−0.125, 0.872]	0.101
IL-17A & SNOT-22	−0.202 [−0.574, 0.290]	0.436	−0.236 [−0.872, 0.503]	0.511	−0.007 [−0.638, 0.693]	0.983
IL-17A & VAS	−0.103 [−0.585, 0.462]	0.693	−0.236 [−0.707, 0.423]	0.533	−0.261 [−0.740, 0.411]	0.413

Serum concentrations are presented as follows: IL-4, IL-5, IL-17A, and IFN-γ in pg/mL; 25(OH)D3 in ng/mL; total IgE in IU/mL; and eosinophil count in cells/µL. LKS, Lund–Kennedy Score; LMS, Lund–Mackay Score. Spearman’s correlation coefficients (ρ [95% BCa CI]) and *p*-values.

## Data Availability

The data that support the findings of this study are available upon request from the corresponding author.

## Abbreviations

The following abbreviations are used in this manuscript:

25(OH)D3	25-hydroxyvitamin D3
CRSwNP	Chronic rhinosinusitis with nasal polyps
CRSsNP	Chronic rhinosinusitis without nasal polyps
CT	Computed tomography
eCRS	eosinophilic chronic rhinosinusitis
IFN-γ	Interferon-gamma
IL	Interleukin
LKS	Lund–Kennedy Score
LMS	Land–Mackay Score
neCRS	Non-eosinophilic chronic rhinosinusitis
NP	Nasal polyp
SNOT-22	Sino-Nasal Outcome Test-22
Th1	Type 1 inflammation
Th2	Type 2 inflammation
Th3	Type 3 inflammation
VDR	Vitamin D receptor

## References

[1] Zand V., Baradaranfar M., Vaziribozorg S., Mandegari M., Mansourimanesh M., Saeidieslami N. (2020). Correlation of Serum Vitamin D Levels with Chronic Rhinosinusitis Disease Severity. Iran. J. Otorhinolaryngol..

[2] Bachert C., Hicks A., Gane S., Peters A.T., Gevaert P., Nash S., Horowitz J.E., Sacks H., Jacob-Nara J.A. (2024). The interleukin-4/interleukin-13 pathway in type 2 inflammation in chronic rhinosinusitis with nasal polyps. Front. Immunol..

[3] Sedaghat A.R., Kuan E.C., Scadding G.K. (2022). Epidemiology of Chronic Rhinosinusitis: Prevalence and Risk Factors. J. Allergy Clin. Immunol. Pract..

[4] Johansson L., Åkerlund A., Melén I., Holmberg K., Bende M. (2003). Prevalence of Nasal Polyps in Adults: The Skovde Population-Based Study. Ann. Otol. Rhinol. Laryngol..

[5] Eschenbacher W., Straesser M., Knoeddler A., Li R.-C., Borish L. (2020). Biologics for the Treatment of Allergic Rhinitis, Chronic Rhinosinusitis, and Nasal Polyposis. Immunol. Allergy Clin. N. Am..

[6] Stevens W.W., Peters A.T., Tan B.K., Klingler A.I., Poposki J.A., Hulse K.E., Grammer L.C., Welch K.C., Smith S.S., Conley D.B. (2019). Associations Between Inflammatory Endotypes and Clinical Presentations in Chronic Rhinosinusitis. J. Allergy Clin. Immunol. Pract..

[7] Benjamin M.R., Stevens W.W., Li N., Bose S., Grammer L.C., Kern R.C., Tan B.K., Conley D.B., Smith S.S., Welch K.C. (2019). Clinical Characteristics of Patients with Chronic Rhinosinusitis without Nasal Polyps in an Academic Setting. J. Allergy Clin. Immunol. Pract..

[8] Tan B.K., Schleimer R.P., Kern R.C. (2010). Perspectives on the etiology of chronic rhinosinusitis. Curr. Opin. Otolaryngol. Head Neck Surg..

[9] Shah S.A., Ishinaga H., Takeuchi K. (2016). Pathogenesis of eosinophilic chronic rhinosinusitis. J. Inflamm..

[10] Bernstein J.A., White A.A., Han J.K., Lang D.M., Elkayam D., Baroody F.M. (2023). Review of evidence supporting the use of nasal corticosteroid irrigation for chronic rhinosinusitis. Ann. Allergy Asthma Immunol..

[11] Bai J., Huang J.H., Price C.P.E., Schauer J.M., Suh L.A., Harmon R., Conley D.B., Welch K.C., Kern R.C., Shintani-Smith S. (2022). Prognostic factors for polyp recurrence in chronic rhinosinusitis with nasal polyps. J. Allergy Clin. Immunol..

[12] Pantazidou G., Papaioannou I., Skoulakis C., Petinaki E., Hajiioannou J. (2023). Vitamin D Levels in Chronic Rhinosinusitis in Patients with or Without Nasal Polyposis: A Systematic Review. Cureus.

[13] Bachert C., Mannent L., Naclerio R.M., Mullol J., Ferguson B.J., Gevaert P., Hellings P., Jiao L., Wang L., Evans R.R. (2016). Effect of Subcutaneous Dupilumab on Nasal Polyp Burden in Patients with Chronic Sinusitis and Nasal Polyposis: A Randomized Clinical Trial. JAMA.

[14] Ryser F.S., Yalamanoglu A., Valaperti A., Brühlmann C., Mauthe T., Traidl S., Soyka M.B., Steiner U.C. (2023). Dupilumab-induced eosinophilia in patients with diffuse type 2 chronic rhinosinusitis. Allergy.

[15] Guo C.-L., Liu F.-F., Wang D.-Y., Liu Z. (2023). Type 2 Biomarkers for the Indication and Response to Biologics in CRSwNP. Curr. Allergy Asthma Rep..

[16] Wang L.-F., Lee C.-H., Chien C.-Y., Chen J.Y.-F., Chiang F.-Y., Tai C.-F. (2013). Serum 25-hydroxyvitamin D levels are lower in chronic rhinosinusitis with nasal polyposis and are correlated with disease severity in Taiwanese patients. Am. J. Rhinol. Allergy.

[17] Kutluğ S., Kılıç M., Bilgici B., Paksu Ş., Yıldıran A., Sancak R. (2017). An evaluation of vitamin D levels in children with seasonal allergic rhinitis during pollen season. Pediatr. Allergy Immunol..

[18] Ismailova A., White J.H. (2022). Vitamin D, infections and immunity. Rev. Endocr. Metab. Disord..

[19] Al-Ebiary H.A., Shafik A.G., Hassan M.A., Taha M.S., El-Naggar Y.M. (2018). Role of vitamin D in chronic rhinosinusitis: A systematic review and meta-analysis study. Egypt. J. Otolaryngol..

[20] Chauss D., Freiwald T., McGregor R., Yan B., Wang L., Nova-Lamperti E., Kumar D., Zhang Z., Teague H., West E.E. (2022). Autocrine vitamin D signaling switches off pro-inflammatory programs of TH1 cells. Nat. Immunol..

[21] Xiong P., Wu H., Wang F., Lu Q., Liu B., Peng S. (2021). The relationship between vitamin D and moderate/severe persistent allergic rhinitis. Rev. Française D’allergologie.

[22] Płudowski P., Ducki C., Konstantynowicz J., Jaworski M. (2016). Vitamin D status in Poland. Pol. Arch. Intern. Med..

[23] Zhou F.W., Zhang T., Jin Y., Ma Y.F., Xian Z.P., He X.C., Wu Z.M., Wang Y., Zhu L., Yuan X.Z. (2021). Predictive diagnostic value of serum 25-hydroxyvitamin D3 in eosinophilic chronic rhinosinusitis with nasal polyps. Chin. J. Otorhinolaryngol. Head Neck Surg..

[24] Bagheri P., Nouri M., Eskandarzadeh H., Darvishi M. (2024). Evaluation of Serum Levels of Vitamin D3 and IgE in Patients with Chronic and Allergic Sinusitis: A Cross-sectional Study. Recent. Adv. Inflamm. Allergy Drug Discov..

[25] Mulligan J.K., Bleier B.S., O’Connell B., Mulligan R.M., Wagner C., Schlosser R.J. (2011). Vitamin D3 correlates inversely with systemic dendritic cell numbers and bone erosion in chronic rhinosinusitis with nasal polyps and allergic fungal rhinosinusitis. Clin. Exp. Immunol..

[26] Konstantinidis I., Fotoulaki M., Iakovou I., Chatziavramidis A., Mpalaris V., Shobat K., Markou K. (2017). Vitamin D_3_ deficiency and its association with nasal polyposis in patients with cystic fibrosis and patients with chronic rhinosinusitis. Am. J. Rhinol. Allergy.

[27] Ali M., Ali O. (2023). Vitamin D deficiency contributes to development of nasal polyps in Iraqi patients suffering from chronic rhinosinusitis. J. Popul. Ther. Clin. Pharmacol..

[28] Mostafa B.E., Taha M.S., Abdel Hamid T., Omran A., Lotfi N. (2016). Evaluation of vitamin D levels in allergic fungal sinusitis, chronic rhinosinusitis, and chronic rhinosinusitis with polyposis. Int. Forum Allergy Rhinol..

[29] Bavi F., Movahed R., Salehi M., Hossaini S., Bakhshaee M. (2019). Chronic rhinosinusitis with polyposis and serum vitamin D levels. Acta Otorhinolaryngol. Ital..

[30] Alharthi G.N.A., Alzarei A. (2024). The Correlation Between Vitamin D Deficiency and Chronic Rhinosinusitis: A Systematic Review. Cureus.

[31] Basha S.I., Hassan A.A., Shaaban M.M., Younis M.A. (2023). Vitamin D3 and chronic rhinosinusitis, Is there a relationship?. Al-Azhar Int. Med. J..

[32] Deluca H.F., Cantorna M.T. (2001). Vitamin D: Its role and uses in immunology. FASEB J..

[33] Ozkara S., Keles E., Ilhan N., Gungor H., Kaygusuz I., Alpay H.C. (2012). The relationship between Th1/Th2 balance and 1α,25-dihydroxyvitamin D3 in patients with nasal polyposis. Eur. Arch. Otorhinolaryngol..

[34] Lee E.J., Hwang C.S., Kim K.-S. (2019). Lack of correlation between serum 25(OH)D level and endoscopy-based chronic rhinosinusitis in Korean adults. Rhinology.

[35] Bai K., Dong H., Liu L., She X., Liu C., Yu M., Liang Z., Lin H., Ke P., Huang X. (2023). Serum 25-hydroxyvitamin D status of a large Chinese population from 30 provinces by LC–MS/MS measurement for consecutive 3 years: Differences by age, sex, season and province. Eur. J. Nutr..

[36] Dupuis M.L., Pagano M.T., Pierdominici M., Ortona E. (2021). The role of vitamin D in autoimmune diseases: Could sex make the difference?. Biol. Sex. Differ..

[37] Singh A., Tekade M.L., Pandey A., Srivastava S., Tyagi A. (2026). Evaluation of Serum Vitamin D Levels in Patients with Chronic Rhinosinusitis. Int. J. Curr. Pharm. Rev. Res..

[38] Ma S.W., Ende J.A., Alvarado R., Christensen J.M., Kalish L., Sacks R., Campbell R., Rimmer J., Harvey R. (2020). Topical Vitamin D May Modulate Human Sinonasal Mucosal Responses to House Dust Mite Antigen. Am. J. Rhinol. Allergy.

[39] Schlosser R.J., Soler Z.M., Schmedes G.W., Storck K., Mulligan J.K. (2014). Impact of vitamin D deficiency upon clinical presentation in nasal polyposis. Int. Forum Allergy Rhinol..

[40] Christensen J.M., Cheng J., Earls P., Gunton J., Sewell W., Sacks R., Harvey R.J. (2017). Vitamin D pathway regulatory genes encoding 1α-hydroxylase and 24-hydroxylase are dysregulated in sinonasal tissue during chronic rhinosinusitis. Int. Forum Allergy Rhinol..

[41] Waldron J.L., Ashby H.L., Cornes M.P., Bechervaise J., Razavi C., Thomas O.L., Chugh S., Deshpande S., Ford C., Gama R. (2013). Vitamin D: A negative acute phase reactant. J. Clin. Pathol..

[42] Cannell J.J., Grant W.B., Holick M.F. (2014). Vitamin D and inflammation. Derm.-Endocrinol..

[43] Feng L., Meng T., Qi Y., Athari S.S., Chen X. (2021). Study Effect of Vitamin D on the Immunopathology Responses of the Bronchi in Murine Model of Asthma. Iran. J. Allergy Asthma Immunol..

[44] Fang X., Xie Q., Zhang X. (2021). Serum vitamin D level in mice with allergic rhinitis is correlated with inflammatory factors. Am. J. Transl. Res..

[45] Yao Y., Xie S., Yang C., Zhang J., Wu X., Sun H. (2017). Biomarkers in the evaluation and management of chronic rhinosinusitis with nasal polyposis. Eur. Arch. Otorhinolaryngol..

[46] Shrestha P., Deepak R., Bhalla A.S., Gupta Y., Sikka K., Irugu D.V.K., Bairwa M., Thakar A., Verma H. (2022). Vitamin D and Interleukins in Chronic Rhinosinusitis with Polyposis. Indian J. Otolaryngol. Head Neck Surg..

[47] Bradley D.T., Kountakis S.E. (2005). Role of Interleukins and Transforming Growth Factor-β in Chronic Rhinosinusitis and Nasal Polyposis. Laryngoscope.

[48] Rai G., Ansari M.A., Dar S.A., Datt S., Gupta N., Sharma S., Haque S., Ramachandran V.G., Mazumdar A., Rudramurthy S. (2018). Serum Cytokine Profile in Patients with Chronic Rhinosinusitis with Nasal Polyposis Infected by *Aspergillus flavus*. Ann. Lab. Med..

[49] Scavuzzo M.C., Fattori B., Ruffoli R., Rocchi V., Carpi A., Berni R., Giambelluca M.A., Giannessi F. (2005). Inflammatory mediators and eosinophilia in atopic and non-atopic patients with nasal polyposis. Biomed. Pharmacother..

[50] Zielińska-Bliźniewska H., Paprocka-Zjawiona M., Merecz-Sadowska A., Zajdel R., Bliźniewska-Kowalska K., Malinowska K. (2022). Serum IL-5, POSTN and IL-33 levels in chronic rhinosinusitis with nasal polyposis correlate with clinical severity. BMC Immunol..

[51] Du K., Huang Z., Si W., Huang Q., Li C., Wang M., Li Y., Wu Y., Qu J., Zhou B. (2020). Dynamic Change of T-Helper Cell Cytokines in Nasal Secretions and Serum after Endoscopic Sinus Surgery in Chronic Rhinosinusitis with Nasal Polyps. ORL.

[52] Tomassen P., Vandeplas G., Van Zele T., Cardell L.-O., Arebro J., Olze H., Förster-Ruhrmann U., Kowalski M.L., Olszewska-Ziąber A., Holtappels G. (2016). Inflammatory endotypes of chronic rhinosinusitis based on cluster analysis of biomarkers. J. Allergy Clin. Immunol..

[53] Kubota K., Takeno S., Taruya T., Sasaki A., Ishino T., Hirakawa K. (2017). IL-5 and IL-6 are increased in the frontal recess of eosinophilic chronic rhinosinusitis patients. J. Otolaryngol. Head Neck Surg..

[54] Aggarwal S., Ghilardi N., Xie M.-H., De Sauvage F.J., Gurney A.L. (2003). Interleukin-23 Promotes a Distinct CD4 T Cell Activation State Characterized by the Production of Interleukin-17. J. Biol. Chem..

[55] Romagnani S., Maggi E., Liotta F., Cosmi L., Annunziato F. (2009). Properties and origin of human Th17 cells. Mol. Immunol..

[56] Zhou L., Lopes J.E., Chong M.M.W., Ivanov I.I., Min R., Victora G.D., Shen Y., Du J., Rubtsov Y.P., Rudensky A.Y. (2008). TGF-β-induced Foxp3 inhibits TH17 cell differentiation by antagonizing RORγt function. Nature.

[57] Klingler A.I., Stevens W.W., Tan B.K., Peters A.T., Poposki J.A., Grammer L.C., Welch K.C., Smith S.S., Conley D.B., Kern R.C. (2021). Mechanisms and biomarkers of inflammatory endotypes in chronic rhinosinusitis without nasal polyps. J. Allergy Clin. Immunol..

[58] Kolls J.K., Lindén A. (2004). Interleukin-17 family members and inflammation. Immunity.

[59] Zielińska-Bliźniewska H., Cuchra-Kulesza M., Nowak-Zduńczyk A., Paprocka-Zjawiona M., Merecz-Sadowska A., Malinowska K. (2024). The role of Th17 lymphocytes in the pathogenesis of chronic rhinosinusitis. Postępy Hig. Med. Doświadczalnej.

[60] Kato A., Schleimer R.P., Bleier B.S. (2022). Mechanisms and pathogenesis of chronic rhinosinusitis. J. Allergy Clin. Immunol..

[61] Ye D., Wang Z., Ye J., Wang M., Liu J., Xu Y., Jiang H., Chen J., Wan J. (2020). Interleukin-5 levels are decreased in the plasma of coronary artery disease patients and inhibit Th1 and Th17 differentiation In Vitro. Rev. Española Cardiol. (Engl. Ed.).

[62] Deng C., Peng N., Tang Y., Yu N., Wang C., Cai X., Zhang L., Hu D., Ciccia F., Lu L. (2021). Roles of IL-25 in Type 2 Inflammation and Autoimmune Pathogenesis. Front. Immunol..

[63] Hu X.-D., Bao Y.-Y., Zhou S.-H., Yao H.-T., Mao J.-Y., Ji X.-X., Wu X.-H. (2013). Interleukin-17A expression in patients with chronic rhinosinusitis and its relationship with clinical features. J. Int. Med. Res..

[64] Hussien H.A., Habieb M.S., Hamdan A.M. (2021). Evaluation of Serum Total Immunoglobulin E, Interleukin-17 and Pentraxin-3 as Biomarkers for Chronic Rhinosinusitis with Nasal Polyposis. Am. J. Rhinol. Allergy.

[65] Hao D., Wu Y., Li P., Li C., Jiang T., Zhang Q., Liu S., Shi L. (2022). An Integrated Analysis of Inflammatory Endotypes and Clinical Characteristics in Chronic Rhinosinusitis with Nasal Polyps. J. Inflamm. Res..

[66] Kim D.W., Eun K.M., Roh E.Y., Shin S., Kim D.-K. (2019). Chronic Rhinosinusitis without Nasal Polyps in Asian Patients Shows Mixed Inflammatory Patterns and Neutrophil-Related Disease Severity. Mediat. Inflamm..

[67] Tan B.K., Klingler A.I., Poposki J.A., Stevens W.W., Peters A.T., Suh L.A., Norton J., Carter R.G., Hulse K.E., Harris K.E. (2017). Heterogeneous inflammatory patterns in chronic rhinosinusitis without nasal polyps in Chicago, Illinois. J. Allergy Clin. Immunol..

[68] Delemarre T., Holtappels G., Ruyck N.D., Zhang N., Nauwynck H., Bachert C., Gevaert E. (2020). Type 2 inflammation in chronic rhinosinusitis without nasal polyps: Another relevant endotype. J. Allergy Clin. Immunol..

[69] Shen Y., Zhang N., Yang Y., Hong S., Bachert C. (2022). Local Immunoglobulin E in nasal polyps: Role and modulation. Front. Immunol..

[70] Chang S.W., Park J.J., Hwang C.S., Nam J.S., Ha J.-G., Almarzouq W.F., Kim C.-H., Yoon J.-H., Cho H.-J. (2021). Role of specific IgE on staphylococcal enterotoxin B in chronic rhinosinusitis severity. Clin. Otolaryngol..

[71] Fokkens W.J., Lund V.J., Hopkins C., Hellings P.W., Kern R., Reitsma S., Toppila-Salmi S., Bernal-Sprekelsen M., Mullol J., Alobid I. (2020). European Position Paper on Rhinosinusitis and Nasal Polyps 2020. Rhinology.

[72] Lund V.J., Mackay I.S. (1993). Staging in rhinosinusitus. Rhinology.

[73] Lund V.J., Kennedy D.W. (1995). Quantification for staging sinusitis. The Staging and Therapy Group. Ann. Otol. Rhinol. Laryngol. Suppl..

[74] Tokunaga T., Sakashita M., Haruna T., Asaka D., Takeno S., Ikeda H., Nakayama T., Seki N., Ito S., Murata J. (2015). Novel scoring system and algorithm for classifying chronic rhinosinusitis: The JESREC Study. Allergy.

